# A Desirable Invention

**Published:** 1868-10

**Authors:** 


*A Desirable Invention*—Dr. W. Leigh Burton, of Richmond, Va., a member of the late graduating class of the Baltimore College of Dental Surgery, has recently been awarded a patent at Washington for an improved electro heating apparatus invented by him.

The primary object of this device is to afford a safe, reliable and economical method of heating railway passenger cars.

The heat is evolved by electrical currents, and radiated from metallic plates. The principle can also be applied to heating residences, public building, etc. It has been submitted to the inspection of several eminent scientific gentlemen, all of whom have expressed the belief that it will accomplish the important result claimed by the inventor.

We feel confident that Dr. Burton's expectations will be fully realized.

